# Supplementary material for: Plant-mediated community structure of spring-fed, coastal rivers
Source: PLoS One. 2019 Dec 30;14(12):e0219236. doi: 10.1371/journal.pone.0219236 (PMC6936880; doi:10.1371/journal.pone.0219236)
Supplement: Attachment — Submitted filename: Ms_Review.pdf [file pone.0219236.s011.pdf]

Comments to the authors by Academic Editor James N. McNair

Ms ID: PONE-D-19-17270
Title: Plant mediated community structure of spring-fed, coastal rivers
Authors: Lauretta M, Pine W, Walters C, Frazer T

This is an interesting manuscript dealing with changes in the structure of fish and invertebrate assemblages in coastal rivers of Florida that are associated with declining abundance of submersed vascular plants and increasing abundance of filamentous macroalgae. The study is based on a large data set for two coastal rivers in Florida and a simulation model. The two rivers differ markedly in the degree to which abundance of submersed vascular plants has declined in the roughly 20 years since the beginning of the data record in 1998. After parameterizing the model mainly with data from the river in which less decline has occurred (the Chassahowitzka River), the model is used to predict changes in the structure of fish and invertebrate assemblages resulting from four alternative scenarios. All the scenarios involve changes in vascular plant and macroalgae abundances (these changes act as forcing functions in the model), and three of them also involve compensatory changes in the search efficiencies of predatory fish in response to declining abundance of vascular plants or increasing abundance of macroalgae or both. The predicted structures of fish and invertebrate assemblages under the four scenarios are compared quantitatively with data from the river that experienced major declines in vascular plant abundance (the Homosassa River). The scenario for which predicted assemblage structures were most similar to the observed structures was identified, and the biological differences between the scenarios were used as the basis for discussing the apparent importance of compensatory changes in search efficiency by predatory fish in these systems as a response to changes in abundance of submersed vascular plants and macroalgae. There is also a good discussion of model limitations due to gaps in current knowledge about some of the major groups of organisms.

Specific comments to the authors follow.

Main comments

1. Line 39: Please delete the word "tests". You do not perform any statistical tests of hypotheses; you simply calculate values of an objective function or goodness-of-fit measure and use those numerical values to compare the prediction accuracy of four alternative scenarios of your ecosystem model.

2. Line 252 and elsewhere: Is it perhaps somewhat misleading to say that you are assessing model fit? Would it perhaps be better to say that you are assessing the accuracy or error of model predictions? For example, in regression analysis, we fit a model to a data set and then assess its fit to the same data. If we have a version of the model with multiple predictor variables and want to find out if some of the predictors can be dropped, then we are doing model selection, but always by comparing each model to the data used to fit it. What you are doing (if I understand it correctly) is different: You are parameterizing a model mainly with data from one system (the Chassahowitzka River) and then checking its predictions under four alternative scenarios with data from a different but related system (the Homosassa River). This is an important difference, and I think you should emphasize it a bit more. The paragraph beginning on line 100 might be a good place to do this by simply modifying the overview of your approach. You want to make sure readers don't think you are doing statistical model selection, and that they realize early on that you are testing predictions on a data set different from the one used to parameterize the model. In my opinion, your approach is stronger than model selection.

3. I have concerns about using Pearson's X^2 statistic as the measure of how well or poorly model predictions match the data for the Homosassa River. (Terminology note: The test statistic used in the chi-squared test is traditionally called X^2 rather than chi-squared or χ^2. For appropriate count data with

c mutually exclusive categories, the distribution of statistic X^2 becomes *approximately* a chi-squared distribution with $c - 1$ degrees of freedom as the sample size becomes large; i.e., X^2 converges in distribution to χ^2_{c-1} as $n \to \infty$.) First of all, this measure is not scale-free, which is a problem here. Because each discrepancy between observed and predicted values is squared but is divided by the non-squared predicted value, the various terms of the two sums in Eq. (1) have the same dimensions as the corresponding predicted (or observed) values. In your model, the biomass terms are on a different scale (dimension = Mass) than the diet-composition terms (these appear to be proportions, which are dimensionless), so it doesn't make sense to add them to form the total X^2 value. (Pearson proposed X^2 as a measure of goodness of fit—it actually measures badness of fit—for multinomial count data, which are dimensionless.) Another problem with X^2 as a measure of how well predictions of a mechanistic model fit data is that squaring the differences between observed and expected values is generally considered to result in undue influence by a small number of unusually large discrepancies between predicted and observed values (for example, this is a common argument in the hydrology literature). Using absolute values of discrepancies instead of squares of discrepancies is the usual way of addressing this issue while still ensuring that all discrepancies are nonnegative and therefore cannot partially cancel when summed.

There is no perfect way to assess model fit or prediction error, because there is always a subjective element involved in deciding how best to compare observed values with values predicted by a model. However, I think you certainly can do better than using X^2 as your measure of overall prediction error. Because you want to combine discrepancies of data types that have different measurement scales, it would be best to rescale the measures of discrepancy somehow so all are dimensionless. The approach you currently use partially rescales the squared discrepancy for each taxon by dividing by the predicted value for that same taxon. A better approach would be to divide each squared discrepancy by the *square* of the predicted value, since each term would then be dimensionless. But in assessing process-based models, it is nonstandard to rescale discrepancies by dividing by predicted values instead of observed values (because the scale for assessing error should be anchored to what actually happened, not what the model predicted). Therefore, I suggest you base your measure of overall discrepancy or prediction error on sums of terms of the form $[(\text{obs} - \text{pred})/\text{obs}]^2 = [1 - (\text{pred}/\text{obs})]^2$ instead of $(\text{obs} - \text{pred})^2/\text{pred}$. Such terms are sometimes called "squared relative errors" in statistics; they make the individual discrepancies or errors dimensionless but still suffer from the problem of inappropriately exaggerating unusually large discrepancies. Better still would be to use terms of the form $|(\text{obs} - \text{pred})/\text{obs}| = |1 - (\text{pred}/\text{obs})|$, sometimes called (somewhat awkwardly) "absolute relative errors" in statistics, which make the individual errors dimensionless while also greatly reducing sensitivity to unusually large deviations. Eq. (1) then becomes

$$E = \sum_{i=1}^{n} \left| 1 - \frac{\text{pred } B_i}{\text{obs } B_i} \right| + \sum_{j=1}^{c} \left| 1 - \frac{\text{pred } DC_j}{\text{obs } DC_j} \right|,$$

where E denotes overall relative prediction error. All terms in both sums are now dimensionless, so there is no problem summing biomass and diet-composition terms. And there is also no problem with a small number of unusually large discrepancies being unduly magnified by squaring them.

This formula, of course, assumes that none of the observed values are zero (because they appear in the denominators). If any are zero, you could use the idea behind the absolute-value form of Willmott's Index of Agreement[1] and use terms of the form $|\text{obs} - \text{pred}|/(\text{obs} + \text{pred})$. Clearly, there should be no taxonomic group for which both observed and predicted values of abundance are zero, since

[1]Legates DR, McCabe GJ. 1999. Evaluating the use of "goodness-of-fit" measures in hydrologic and hydroclimatic model validation. *Water Resources Research* **35**: 233–241.

your implementation of X^2 requires that all predicted values be positive. Alternatively, you could use the full-blown absolute-value form of Willmott's index (with biomass and diet-composition terms in separate sums), but that would involve a weaker measure of prediction error because errors for each taxon would be scaled to the average discrepancy over all taxa instead of being scaled to values for the same taxon.

4. A general concern I have about your approach of comparing predictions of the four scenarios with data for the Homosassa River is that scenarios 2–4 appear to be more complex than scenario 1, and scenario 4 appears to be more complex than scenarios 2 and 3. Good model-selection methods like the AIC approach that Burnham & Anderson advocate penalize complexity as a way to balance the ability of models with more parameters to fit complicated patterns better than models with fewer parameters for mathematical rather than biological reasons. Because predictions of your most complex scenario appear to match the data for the Homosassa River better than those of the simpler scenarios, the question arises as to whether this is surprising or even meaningful. But I think this criticism does not apply to your approach, because your scenarios appear to have been formulated *a priori* rather than by fitting the model to the data. I suggest you give some thought to discussing this issue in the Discussion, since readers familiar with good model selection procedures might wonder about it.

5. Table 6: You currently do not explain how you calculated the entries in this table other than the totals for the four scenarios. I assume the other entries correspond to individual terms of the sums in Eq. (1), but I'm not certain that assumption is correct. A brief explanation needs to be included somewhere, and I think the best place is in the table caption.

Other comments

1. Lines 93–94: Please revise the phrase "necessitating a need for science to inform restoration strategies". Certainly the word "necessitating" should be changed; I suggest replacing it with "creating". I interpret the rest of this phrase as saying that the current knowledge gap regarding causal mechanisms of vascular plant loss creates a need for *new* science. If my interpretation is correct, I suggest inserting the word "new" between "for" and "science" in line 94.

2. Line 108: Please change "hypotheses" to "scenarios" so the text here matches your subsequent description in the Methods section more closely. This change will also help readers avoid the mistake of thinking that you are going to test statistical hypotheses.

3. Table 2: Please define P/B and Q/B (or P, B, and Q) in the table caption. You mention in the text (line 352) that P denotes production and B denotes biomass, but I don't see any statement as to what Q denotes.

4. Line 191: Please insert a right parenthesis after "[35]".

5. Lines 204–205: I think you need to provide a brief description of the Ecopath/Ecosim model. Most readers (including myself) will not be familiar with it, but some familiarity seems necessary in order to adequately understand how you parameterized and used the model.

6. Table 4: Please define P/B and Q/B in the table caption, or state that they are defined as in Table 2.

7. Figure 3: Please change "Model" to "Scenario" in the column headings. The text consistently refers to these four combinations of forcing and mediation functions as scenarios, so the same term should be used in this figure.

8. Line 241–251: I think this text will be clearer if you devote one paragraph to each scenario, even though that means there will be only one sentence in some of the paragraphs. So, start a new paragraph in line 244, beginning with “Scenario 3”. Then change the beginning of current line 248 from “Scenario 3” to “This scenario” and join this line to the end of the previous one, yielding one paragraph entirely about Scenario 3. Then start a new paragraph in current line 249, beginning with “Scenario 4”.

9. Line 244: I suggest you begin this sentence as follows: “In contrast to scenario 2, scenario 3...”. The idea is to immediately indicate to the reader that you are specifying how scenario 3 differs from scenario 2.

10. Lines 249–250: I suggest you change this sentence to read as follows: “Scenario 4 included both the mediation function between vascular plant and large-bodied fishes of scenario 2 and the mediation function between filamentous macroalgae and small-bodied fishes of scenario 3 (Fig. 3).”

11. Line 306: Please delete “vegetative” or replace with a better word.

12. Table 6: Please change “Chi-squared (X^2) statistics...” to “Goodness-of-fit measures...” or, better still, “Prediction errors”. In statistics, “Chi-squared” refers to a specific probability distribution or a formal statistical test based on a test statistic with that distribution, whereas you are simply giving numerical values for an objective function, or goodness-of-fit function, for the four alternative scenarios of your ecosystem model. Also, in statistics, the term “statistic” refers to a function of random variables that has a well-defined distribution and whose value is fixed by the observations in a sample. It would be better to use the term “measure” here, or the term “Prediction errors”, because you do not know or make any use of the distribution of your measure of goodness of fit (or prediction error); you simply use it as a relative measure of prediction error under the four alternative scenarios.

13. Table 6: Please change “Model” to “Scenario” in the column headings, for the same reason stated above in my comment about Fig. 3. In addition to the disconnect between terminology in the text and this table, using the term “model” here, coupled with your use of the term “chi-squared” in the table caption, suggests you are performing statistical model selection (which would require a procedure based on p values or a suitable form of AIC), but that is not what you are doing.

14. Table 6: Please remove “(Obs−Pred)^2” from the heading of column 1, since that type of information does not appear in this column. When you revise the table caption to address my comment above, the caption will fully explain what the various numerical values represent, so there is no need to repeat that explanation in the table itself. If you nevertheless feel this will be useful for readers, you will need to create an additional heading in the table that spans columns 2 through 5 and says something like “Goodness-of-fit measure” or “Prediction error”. Since this measure should be dimensionless (as I noted above), there will be no measurement units to be specify.

15. Line 402: Please change “macroalgae provides” to “macroalgae appear to provide”.

16. Lines 411–412: The word “support” is used in two different ways in this part of the sentence. For clarity, I suggest you revise it along the following lines: “Our results also support the hypothesis that high plant densities adversely affect the feeding efficiency of fishes and help sustain high densities of prey taxa...”

17. Line 423: Please insert “Camp et al.” before “[26]”. Also, in reference [26], change “PINE” to “Pine”.

18. Line 426: Please insert “Kornijów et al.” before “[44]”.

19. Line 440: Please change “dynamics” to “interactions”; “predator-prey dynamics” typically refers to population dynamics rather than the sort of compensatory foraging behavior you seem to refer to here.
20. Lines 429–457: Excellent discussion of model shortcomings and how they are related to gaps in current knowledge that need to be filled.
21. Line 480: Please insert “Bettoli et al.” before “[46]”.
22. Line 494: Please insert “Heffernan et al.” before “[18]”.
23. Line 497: Please insert “Wood et al.” before “[45]”.
