# Supplementary material for: Plant-mediated community structure of spring-fed, coastal rivers
Source: PLoS One. 2019 Dec 30;14(12):e0219236. doi: 10.1371/journal.pone.0219236 (PMC6936880; doi:10.1371/journal.pone.0219236)
Supplement: Attachment — Submitted filename: Response to reviewers.docx [file pone.0219236.s012.docx]

PONE-D-19-17270
Plant mediated community structure of spring-fed, coastal rivers
PLOS ONE

RESPONSE TO REVIEWERS AND FORMAT REQUIREMENTS

1. As I explain in my review, I think it is important that you modify your measure of prediction error (or goodness of fit) to make it dimensionless, since terms in the current X^2 formula do not share the same physical dimensions and therefore should not be added. I suggest a simple alternative in my review. This change requires recalculation of the values listed in Table 6, but I will be surprised if it changes your conclusions.

Author response: Thanks for highlighting this discrepancy in the model evaluation versus statistical goodness-of-fit. The manuscript was revised throughout to eliminate all references to statistical tests, goodness-of-fit, as well as chi-squared statistic. The revisions are documented below under the editor’s review comments. Note that the model prediction accuracy was calculated as absolute error, which retained the dimensions of biomass. This was deemed to be important in order to demonstrate the ecosystem model capability to predict the community structure under the different scenarios, and retain the scale of trophic group biomass changes. That is, we want to demonstrate the scale of observed versus predicted biomass, as some trophic groups comprise major biomass components versus ones that were observed in small numbers/biomass. Also note we removed the sum value of the biomass and diet composition error combined, since as pointed out, they have different scales and therefore represent different metrics. We also discuss these separately in the results and discussion, rather than as an overall model error term.

2. Please clarify in the text that you are not performing statistical model selection or statistical goodness-of-fit assessments, and therefore no formal tests of statistical significance are performed. Both Reviewer 1 and I were initially puzzled by the way you currently describe your measure of goodness of fit. By calling it "chi-squared" and stating in the abstract that you perform "goodness-of-fit tests", you suggest to readers that you are performing chi-squared tests of statistical significance, but you actually do not do that.

Author response: see comment above and in response to reviewer and editor’s review comments with line references below.

3. Please be sure to address the question by Reviewer 1 regarding availability of all data from your study.

Author response: all datasets from the intensive field surveys have been uploaded as supplementary information. Also note that a Data Dictionary is provided that defines data field headers, describes the metrics listed, and provided a definition of all species codes and other metrics used in the databases.

To enhance the reproducibility of your results, we recommend that if applicable you deposit your laboratory protocols in [protocols.io](http://protocols.io), where a protocol can be assigned its own identifier (DOI) such that it can be cited independently in the future. For instructions see: <http://journals.plos.org/plosone/s/submission-guidelines#loc-laboratory-protocols>

Author response: The field and lab protocols are documented in detail in companion papers referenced in the Methods and materials section.

Journal Requirements:

When submitting your revision, we need you to address these additional requirements.

1. Please ensure that your manuscript meets PLOS ONE's style requirements, including those for file naming. The PLOS ONE style templates can be found at

Author response: The manuscript has been reformatted following the posted guidelines.

2. In your Methods section, please provide additional location information of the study area, including geographic coordinates for the data set if available.

Author response: A table was added (now Table 1), listing the upstream and downstream coordinates of each reach sampled in the rivers, and table reference provided in lines 180-181 of the manuscript with track changes.

3. To comply with PLOS ONE submissions requirements, please provide methods of sacrifice in the Methods section of your manuscript.

Author response: Methods of diet collection were added to distinguish which trophic groups were sampled live versus sacrificed for dissection, and the method of sacrifice listed (lines 192-196 of the document with track changes).

4. We note that you have indicated that data from this study are available upon request. PLOS only allows data to be available upon request if there are legal or ethical restrictions on sharing data publicly. For more information on unacceptable data access restrictions, please see <http://journals.plos.org/plosone/s/data-availability#loc-unacceptable-data-access-restrictions>.

Author response: All datasets are provided to be included as supporting information, as well as the Ecopath with Ecosim model of the Chassahowitzka River, for transparency in the findings and reproducibility of results.

In your revised cover letter, please address the following prompts:

a) If there are ethical or legal restrictions on sharing a de-identified data set, please explain them in detail (e.g., data contain potentially sensitive information, data are owned by a third-party organization, etc.) and who has imposed them (e.g., an ethics committee). Please also provide contact information for a data access committee, ethics committee, or other institutional body to which data requests may be sent.

Author response: A sentence has been included in the cover letter to indicate that no ethical or legal restrictions to sharing the data have been identified.

b) If there are no restrictions, please upload the minimal anonymized data set necessary to replicate your study findings as either Supporting Information files or to a stable, public repository and provide us with the relevant URLs, DOIs, or accession numbers. For a list of acceptable repositories, please see <http://journals.plos.org/plosone/s/data-availability#loc-recommended-repositories>.

Author response: All databases are provided as Supporting Information files, as well as a Data Dictionary describing in detail the contents of each data file.

We will update your Data Availability statement on your behalf to reflect the information you provide.

Author response: Thanks.

Reviewers' comments:

Reviewer's Responses to Questions

**Comments to the Author**

1. Is the manuscript technically sound, and do the data support the conclusions?

The manuscript must describe a technically sound piece of scientific research with data that supports the conclusions. Experiments must have been conducted rigorously, with appropriate controls, replication, and sample sizes. The conclusions must be drawn appropriately based on the data presented.

Reviewer #1: Partly

2. Has the statistical analysis been performed appropriately and rigorously?

Reviewer #1: Yes

3. Have the authors made all data underlying the findings in their manuscript fully available?

The [PLOS Data policy](http://www.plosone.org/static/policies.action#sharing) requires authors to make all data underlying the findings described in their manuscript fully available without restriction, with rare exception (please refer to the Data Availability Statement in the manuscript PDF file). The data should be provided as part of the manuscript or its supporting information, or deposited to a public repository. For example, in addition to summary statistics, the data points behind means, medians and variance measures should be available. If there are restrictions on publicly sharing data—e.g. participant privacy or use of data from a third party—those must be specified.

Reviewer #1: No

Author response: All datasets are now provided in entirety.

 4. Is the manuscript presented in an intelligible fashion and written in standard English?

PLOS ONE does not copyedit accepted manuscripts, so the language in submitted articles must be clear, correct, and unambiguous. Any typographical or grammatical errors should be corrected at revision, so please note any specific errors here.

Reviewer #1: Yes

5. Review Comments to the Author

Please use the space provided to explain your answers to the questions above. You may also include additional comments for the author, including concerns about dual publication, research ethics, or publication ethics. (Please upload your review as an attachment if it exceeds 20,000 characters)

Reviewer #1: The manuscript appeared to be technically sound with the overall conclusions supported by the modeling results. Nevertheless, I did have two questions regarding the interpretation of results.

(a) Is it problematic that none of the models seem to be similar to the Chassahowitka River? Why were the results of Model 1 not more similar to the Chassahowitka River (see Fig. 6)? (

Author response: No, all model scenarios are projections of the future state of the ecosystem under continued plant loss and replacement by filamentous algae. Therefore, it is expected that the community structure will diverge from the highly vegetated state. The strength is in the comparison to the community structure of the adjacent river that has undergone the changes in producer community (i.e. the Homosassa).

c) From a philosophical perspective, what if all the models you tested were "bad"? How would those results differ from what was found? The approach seems to evaluate which is the best model among the ones tested, but how do we know if any of the models are "good"?

Author response: The goal was to produce an ecosystem model with the best predictive capability of community structure under altered producer regimes. The measure of how good a model performs is how the predictions compare to the system with the observed changes in producers. We highlight and discuss in detail which trophic groups are accurately predicted versus those where the predictions diverge considerably from the model scenarios, as well as provide hypotheses of why these diverges may have occurred.

The Ecopath/Ecosim modeling seemed appropriate as well as the approach used to evaluate competing models. I initially expected AIC to be used to evaluate models, but it is not straightforward to me how such an approach could be implemented given the structure of the models being evaluated. Some clarifications are warranted, especially for readers that do not have a deep understand of Ecopath/Ecosim.

Author response: A brief description of the Ecopath and Ecosim model frameworks are provided in lines 221-225 and 240-243. A discussion of model selection approach was added in lines 460-464.

(a) P/B and Q/B ratios should be clearly defined in Table 2.

Author response: revision made

(b) Make sure Walters et al. (2008), which is cited in Table 2, is listed in the references.

Author response: revision made. Also note the addition of all references in Table 2 to the Literature Cited, which were unintentionally left out. All references were renumbered in order of appearance as well.

(c) Provide more context for interpreting the Chi-squared statistics reported in Table 6. What do small and large values indicate? What is the null hypothesis underlying the Chi-squared statistic? Is there a way to calculate p-values for the total Chi-squared statistics? How common is this type of approach (i.e., using Chi-squared statistics) for evaluating these types of models?

Author response: The error measurement was revised as discussed in the above responses, simplified to the absolute prediction error. This should greatly increase the interpretation and eliminate the need for a formal hypothesis test. The objective at the end of the day is to best predict expected changes in river communities under plant loss and replacement of filamentous algae. Certainly this is a goal that does not end at this research, but rather this is a fundamental step in producing mechanistic quantitative models of ecosystem structure and function.

(d) Can you provide context for why a bootstrapping procedure was used for estimating mean trophic group biomass (Lines 167-168)?

Author response: bootstrapping was used to estimate mean biomasses by trophic group because it is robust to distributional assumptions of the data. Further, because of the large number of trophic groups estimated, the bootstrap method provides an efficient approach to estimating means, rather than distribution fitting to each of the trophic group biomass estimates across sample periods.

The data from the modeling output do not seem to be fully available. If this is the case, then this should be easily addressed by providing the output in supporting information.

Author response: Suggestion taken. All data are provided in entirety as supporting information, as well as the parameterized Ecopath model of the Chassahowitzka River for replication of findings and additional hypothesis exploration by interested researchers.

Overall, the manuscript was well written and easy to follow.

Author response: I appreciate the feedback.

The quality of Figures 4, 6, and 7 made interpretation difficult, but this was likely the result of the file I printed.

Author response: All figures have now been quality assured by using the Apex.PACE application, as required by the Editorial team.

6. PLOS authors have the option to publish the peer review history of their article ([what does this mean?](https://journals.plos.org/plosone/s/editorial-and-peer-review-process#loc-peer-review-history)). If published, this will include your full peer review and any attached files.


If you choose “no”, your identity will remain anonymous but your review may still be made public.


**Do you want your identity to be public for this peer review?** For information about this choice, including consent withdrawal, please see our [Privacy Policy](https://www.plos.org/privacy-policy).

Reviewer #1: No


[NOTE: If reviewer comments were submitted as an attachment file, they will be attached to this email and accessible via the submission site. Please log into your account, locate the manuscript record, and check for the action link "View Attachments". If this link does not appear, there are no attachment files to be viewed.]


While revising your submission, please upload your figure files to the Preflight Analysis and Conversion Engine (PACE) digital diagnostic tool, <https://pacev2.apexcovantage.com/>. PACE helps ensure that figures meet PLOS requirements. To use PACE, you must first register as a user. Registration is free. Then, login and navigate to the UPLOAD tab, where you will find detailed instructions on how to use the tool. If you encounter any issues or have any questions when using PACE, please email us at [figures@plos.org](mailto:figures@plos.org). Please note that Supporting Information files do not need this step.

Author response: All figures files were uploaded to PACE and the reformatted Figures are provided.

# Main comments

1. Line 39: Please delete the word “tests”. You do not perform any statistical tests of hypotheses; you simply calculate values of an objective function or goodness-of-fit measure and use those numerical values to compare the prediction accuracy of four alternative scenarios of your ecosystem model.

Author response: revision made

2. Line 252 and elsewhere: Is it perhaps somewhat misleading to say that you are assessing model fit? Would it perhaps be better to say that you are assessing the accuracy or error of model predictions? For example, in regression analysis, we fit a model to a data set and then assess its fit to the same	data. If we have a version of the model with multiple predictor variables and want to find out if some	of the predictors can be dropped, then we are doing model selection, but always by comparing each model to the data used to fit it. What you are doing (if I understand it correctly) is different: You	are parameterizing a model mainly with data from one system (the Chassahowitzka River) and then checking its predictions under four alternative scenarios with data from a different but related system (the Homosassa River). This is an important difference, and I think you should emphasize it a bit	more. The paragraph beginning on line 100 might be a good place to do this by simply modifying the overview of your approach. You want to make sure readers don’t think you are doing statistical model selection, and that they realize early on that you are testing predictions on a data set different from the one used to parameterize the model. In my opinion, your approach is stronger than model selection.

Author response: Extensive revision made to address this issue, as discussed above. All model selection metrics are now estimated as absolute prediction error (|obs-pred|). This seemed the most appropriate, given our objective of producing the best model to predict community structure under alternative producer regimes.

1. I have concerns about using Pearson’s *X*2 statistic as the measure of how well or poorly model predic- tions match the data for the Homosassa River. (Terminology note: The test statistic used in the chi- squared test is traditionally called *X*2 rather than chi-squared or *χ*^2^. For appropriate count data with

*c* mutually exclusive categories, the distribution of statistic *X*2 becomes *approximately* a chi-squared distribution with *c* − 1 degrees of freedom as the sample size becomes large; i.e., *X*2 converges in

distribution to *χ*^2^	as *n* → ∞.) First of all, this measure is not scale-free, which is a problem here.

Because each

*c*−1

y between observed and predicted values is squared but is divided by the

discrepanc

non-squared predicted value, the various terms of the two sums in Eq. (1) have the same dimensions as the corresponding predicted (or observed) values. In your model, the biomass terms are on a differ- ent scale (dimension = Mass) than the diet-composition terms (these appear to be proportions, which are dimensionless), so it doesn’t make sense to add them to form the total *X*2 value. (Pearson pro- posed *X*2 as a measure of goodness of fit—it actually measures badness of fit—for multinomial count data, which are dimensionless.) Another problem with *X*2 as a measure of how well predictions of a mechanistic model fit data is that squaring the differences between observed and expected values is generally considered to result in undue influence by a small number of unusually large discrepancies between predicted and observed values (for example, this is a common argument in the hydrology literature). Using absolute values of discrepancies instead of squares of discrepancies is the usual way of addressing this issue while still ensuring that all discrepancies are nonnegative and therefore cannot partially cancel when summed.

There is no perfect way to assess model fit or prediction error, because there is always a subjective element involved in deciding how best to compare observed values with values predicted by a model. However, I think you certainly can do better than using *X*2 as your measure of overall prediction error. Because you want to combine discrepancies of data types that have different measurement scales, it would be best to rescale the measures of discrepancy somehow so all are dimensionless. The approach you currently use partially rescales the squared discrepancy for each taxon by dividing by the predicted value for that same taxon. A better approach would be to divide each squared discrepancy by the *square* of the predicted value, since each term would then be dimensionless. But in assessing process- based models, it is nonstandard to rescale discrepancies by dividing by predicted values instead of observed values (because the scale for assessing error should be anchored to what actually happened, not what the model predicted). Therefore, I suggest you base your measure of overall discrepancy or prediction error on sums of terms of the form [(obs pred)*/*obs]^2^ = [1 (pred*/*obs)]2 instead of (obs pred)^2^*/*pred. Such terms are sometimes called “squared relative errors” in statistics; they make the individual discrepancies or errors dimensionless but still suffer from the problem of inappropriately exaggerating unusually large discrepancies. Better still would be to use terms of the form (obs pred)*/*obs = 1	(pred*/*obs) , sometimes called (somewhat awkwardly) “absolute relative errors” in statistics, which make the individual errors dimensionless while also greatly reducing sensitivity to unusually large deviations. Eq. (1) then becomes

−	−	−

| | − |

| −

*c*

*n*

*E* =

\'

*i*=1

1 −

pred *Bi* obs *Bi*

\'*j*=1

1 −

pred *DCj ,*

obs *DCj*

where *E* denotes overall relative prediction error. All terms in both sums are now dimensionless, so there is no problem summing biomass and diet-composition terms. And there is also no problem with a small number of unusually large discrepancies being unduly magnified by squaring them.

+

This formula, of course, assumes that none of the observed values are zero (because they appear in the denominators). If any are zero, you could use the idea behind the absolute-value form of Willmott’s Index of Agreement1 and use terms of the form obs pred */*(obs + pred). Clearly, there should be no taxonomic group for which both observed and predicted values of abundance are zero, since

| − |

^1^Legates DR, McCabe GJ. 1999. Evaluating the use of “goodness-of-fit” measures in hydrologic and hydroclimatic model validation. *Water Resources Research* **35**: 233–241.

your implementation of *X*2 requires that all predicted values be positive. Alternatively, you could use the full-blown absolute-value form of Willmott’s index (with biomass and diet-composition terms in separate sums), but that would involve a weaker measure of prediction error because errors for each taxon would be scaled to the average discrepancy over all taxa instead of being scaled to values for the same taxon.

Author response: Point well taken, and addressed by removing reference to goodness-of-fit test statistics and changes the metric and text to prediction error measured as absolute error in biomass. Additionally, the biomass and diet composition errors are no longer summed together, since the dimensions of scale are different. The rationale for keeping scale in the metric is discussed prior.

3. A general concern I have about your approach of comparing predictions of the four scenarios with	data for the Homosassa River is that scenarios 2–4 appear to be more complex than scenario 1, and scenario 4 appears to be more complex than scenarios 2 and 3. Good model-selection methods like the AIC approach that Burnham & Anderson advocate penalize complexity as a way to balance the ability of models with more parameters to fit complicated patterns better than models with fewer parameters for mathematical rather than biological reasons. Because predictions of your most complex scenario appear to match the data for the Homosassa River better than those of the simpler scenarios, the question arises as to whether this is surprising or even meaningful. But I think this criticism does	not apply to your approach, because your scenarios appear to have been formulated *a priori* rather	than by fitting the model to the data. I suggest you give some thought to discussing this issue in the Discussion, since readers familiar with good model selection procedures might wonder about it.

Author response: I also gave considerable thought to the model selection, and typically prefer the approaches outlined by Burnham and Anderson. It wasn’t straight-forward on how this could be implemented, and at the end of the day, it seemed most appropriate to select the best model at predicting community biomass structure, as this is the primary concern of resource managers. However, we don’t indicate that one model is the overall best, but now present the results and discuss the strengths and weaknesses in detail. I also added text in lines 460-465 of the Discussion with the basis for model selection.

4. Table 6: You currently do not explain how you calculated the entries in this table other than the totals for the four scenarios. I assume the other entries correspond to individual terms of the sums in Eq. (1), but I’m not certain that assumption is correct. A brief explanation needs to be included somewhere, and I think the best place is in the table caption.

Author response: revision made in the table caption to indicate absolute error in obs-pred.

# Other comments

1. Lines 93–94: Please revise the phrase “necessitating a need for science to inform restoration strate- gies”. Certainly the word “necessitating” should be changed; I suggest replacing it with “creating”. I interpret the rest of this phrase as saying that the current knowledge gap regarding causal mechanisms of vascular plant loss creates a need for *new* science. If my interpretation is correct, I suggest inserting the word “new” between “for” and “science” in line 94.

Author response: revision made

2. Line 108: Please change “hypotheses” to “scenarios” so the text here matches your subsequent de- scription in the Methods section more closely. This change will also help readers avoid the mistake of thinking that you are going to test statistical hypotheses.

Author response: revision made. Also note the revision to Figs 6 and 7 for consistency in reference to model projection “Scenarios” rather than hypotheses or separate ecosystem models.

3. Table 2: Please define P/B and Q/B (or P, B, and Q) in the table caption. You mention in the text (line

352) that P denotes production and B denotes biomass, but I don’t see any statement as to what Q denotes.

Author response: revision made

4. Line 191: Please insert a right parenthesis after “[35]”.

Author response: revision made

5. Lines 204–205: I think you need to provide a brief description of the Ecopath/Ecosim model. Most readers (including myself) will not be familiar with it, but some familiarity seems necessary in order to adequately understand how you parameterized and used the model.

Author response: A brief description of the Ecopath and Ecosim model frameworks are provided in lines 221-225 and 240-243 of the Methods.

6. Table 4: Please define P/B and Q/B in the table caption, or state that they are defined as in Table 2.

Author response: revision made

7. Figure 3: Please change “Model” to “Scenario” in the column headings. The text consistently refers	to these four combinations of forcing and mediation functions as scenarios, so the same term should	be used in this figure.

Author response: revision made to all table and figures to refer to the alternative projections as Scenarios rather than individual models.

Line 241–251: I think this text will be clearer if you devote one paragraph to each scenario, even though that means there will be only one sentence in some of the paragraphs. So, start a new para- graph in line 244, beginning with “Scenario 3”. Then change the beginning of current line 248 from “Scenario 3” to “This scenario” and join this line to the end of the previous one, yielding one para- graph entirely about Scenario 3. Then start a new paragraph in current line 249, beginning with “Scenario 4”.

Author response: revision made

8. Line 244: I suggest you begin this sentence as follows: “In contrast to scenario 2, scenario 3...”. The idea is to immediately indicate to the reader that you are specifying how scenario 3 differs from scenario 2.

Author response: revision made

9. Lines 249–250: I suggest you change this sentence to read as follows: “Scenario 4 included both the mediation function between vascular plant and large-bodied fishes of scenario 2 and the mediation function between filamentous macroalgae and small-bodied fishes of scenario 3 (Fig. 3).”

Author response: revision made

10. Line 306: Please delete “vegetative” or replace with a better word.

Author response: revision made

11. Table 6: Please change “Chi-squared (*X*^2^) statistics...” to “Goodness-of-fit measures...” or, better still, “Prediction errors”. In statistics, “Chi-squared” refers to a specific probability distribution or a formal statistical test based on a test statistic with that distribution, whereas you are simply giving numerical values for an objective function, or goodness-of-fit function, for the four alternative scenarios of your ecosystem model. Also, in statistics, the term “statistic” refers to a function of random variables that	has a well-defined distribution and whose value is fixed by the observations in a sample. It would be better to use the term “measure” here, or the term “Prediction errors”, because you do not know or	make any use of the distribution of your measure of goodness of fit (or prediction error); you simply	use it as a relative measure of prediction error under the four alternative scenarios.

Author response: revision made , the term prediction error is used in preference to goodness-of-fit for the reasons outlined by the editor and discussed above.

12. Table 6: Please change “Model” to “Scenario” in the column headings, for the same reason stated	above in my comment about Fig. 3. In addition to the disconnect between terminology in the text and this table, using the term “model” here, coupled with your use of the term “chi-squared” in the table caption, suggests you are performing statistical model selection (which would require a procedure	based on *p* values or a suitable form of AIC), but that is not what you are doing.

Author response: revisions made , as well as Figs 6 and 7

13. Table 6: Please remove “(Obs	Pred)^2^” from the heading of column 1, since that type of information does not appear in this column. When you revise the table caption to address my comment above, the caption will fully explain what the various numerical values represent, so there is no need to repeat	that explanation in the table itself. If you nevertheless feel this will be useful for readers, you will	need to create an additional heading in the table that spans columns 2 through 5 and says something	like “Goodness-of-fit measure” or “Prediction error”. Since this measure should be dimensionless (as	I noted above), there will be no measurement units to be specify.

−

Author response: revision made and discussed above.

14. Line 402: Please change “macroalgae provides” to “macroalgae appear to provide”.

Author response: revision made

15. Lines 411–412: The word “support” is used in two different ways in this part of the sentence. For clarity, I suggest you revise it along the following lines: “Our results also support the hypothesis that high plant densities adversely affect the feeding efficiency of fishes and help sustain high densities of prey taxa...”

Author response: revision made

16. Line 423: Please insert “Camp et al.” before “[26]”. Also, in reference [26], change “PINE” to “Pine”.
17. Author response: revision made
18. Line 426: Please insert “Kornijo´ w et al.” before “[44]”.

Author response: revision made

19. Line 440: Please change “dynamics” to “interactions”; “predator-prey dynamics” typically refers to population dynamics rather than the sort of compensatory foraging behavior you seem to refer to here.

Author response: revision made

20. Lines 429–457: Excellent discussion of model shortcomings and how they are related to gaps in current knowledge that need to be filled.

Author response: I appreciate the feedback. In the end, this research is major advancement in the ability to quantify ecosystem structure and mechanisms of fish and invertebrate community responses to producer dynamics, but certainly not an endpoint of our understanding of ecosystem drivers.

21. Line 480: Please insert “Bettoli et al.” before “[46]”.

Author response: revision made

22. Line 494: Please insert “Heffernan et al.” before “[18]”.

Author response: revision made

23. Line 497: Please insert “Wood et al.” before “[45]”.

Author response: revision made
